# Supplementary material for: End Sequence Analysis Toolkit (ESAT) expands the extractable information from single-cell RNA-seq data
Source: Genome Res. 2016 Oct;26(10):1397–410. doi: 10.1101/gr.207902.116 (PMC5052061; doi:10.1101/gr.207902.116)
Supplement: Supplemental Material [file supp_gr.207902.116_Supp_Materials.tar.gz › ESAT-esat_v0.1/doc/html/annotated.html]

ESAT: Class List


ESAT


- Main Page
- Related Pages
- Packages
- Classes
- Files

- Class List
- Class Index
- Class Hierarchy
- Class Members


All Classes Namespaces Files Functions Variables Enumerator Pages

Class List

Here are the classes, structs, unions and interfaces with brief descriptions:

[detail level 123456]

|  |  |
| --- | --- |
| ▼Nbroad |  |
| ▼Ncore |  |
| ►Nannotation |  |
| CAnnotationFactory< T extends LightweightGenomicAnnotation > |  |
| CAnnotationFactoryFactory |  |
| CAnnotationHandler |  |
| CAnnotationReader< T extends GenomicAnnotation > |  |
| CAnnotationReaderFactory |  |
| CAnnotationUtils |  |
| CBasicAnnotationReader |  |
| CBasicGenomicAnnotation |  |
| CBasicGenomicAnnotationReader |  |
| CBasicLightweightAnnotation |  |
| CBasicTwoSubjectAnnotation |  |
| CBED |  |
| CBEDGraph |  |
| CBEDGraphReader |  |
| CBEDReader |  |
| CFeature |  |
| CGenomicAnnotation |  |
| CGenomicAnnotationFilter< T extends LightweightGenomicAnnotation > |  |
| CGFF |  |
| CGFFReader |  |
| CLightweightGenomicAnnotation |  |
| CMaximumContiguousSubsequence |  |
| CRepeatMaskerAnnotation |  |
| CRepeatMaskerAnnotationReader |  |
| CShortBED |  |
| CShortBEDReader |  |
| CTwoSubjectAnnotation |  |
| ►Ndatastructures |  |
| CGenericRootedTree< T > |  |
| CIllegalTreeOperationException |  |
| CInterval |  |
| ►CIntervalTree< V > |  |
| CFwdIterator |  |
| COverlapIterator |  |
| CRevIterator |  |
| ►CJCSCache |  |
| CJCSNodeIterator |  |
| CMatrixWithHeaders |  |
| CMatrixWithHeaderUtils |  |
| CPair< T1 > |  |
| CReversibleIterator< E > |  |
| ►Nerror |  |
| CParseException |  |
| ►Nhmm |  |
| CBadModelException |  |
| CMarkovModel< T > |  |
| CMarkovState< T > |  |
| ►Nmath |  |
| CANOVA |  |
| CCombinationGenerator |  |
| CComputeFDR |  |
| CDistribution |  |
| CEmpiricalDistribution |  |
| CFDRDistribution |  |
| CFisherCombinedProbabilityTest |  |
| CKSTest |  |
| CLoess |  |
| CMannWhitney |  |
| CMannWhitneyPermutations |  |
| CMathUtil |  |
| CPermutationDistribution |  |
| CScanStatistics |  |
| CStatistics |  |
| CUnfairDie |  |
| CWilcox2Sample |  |
| ►Nmotif |  |
| ►Nmeme |  |
| CAMEResultReader |  |
| CMemeMotif |  |
| CMemeResultReader |  |
| ►Nmisc |  |
| CComputeConservedMotifs |  |
| CScoreMotifInstances |  |
| CPositionWeightColumn |  |
| CPositionWeightMatrix |  |
| CPositionWeightMatrixIO |  |
| CPWMUtils |  |
| CSearchException |  |
| CSequenceMotif |  |
| CSequenceMotifCluster |  |
| ►Nmultiplealignment |  |
| CAlignmentExtractor |  |
| CFastaMultipleAlignmentIO |  |
| CMAFAlignment |  |
| CMAFIO |  |
| CMultipleAlignment |  |
| CMultipleAlignmentFactory |  |
| CMultipleAlignmentIO |  |
| CMultipleAlignmentIOFactory |  |
| CPhylipInterleavedMultipleAlignmentIO |  |
| CPhylipSequencialMultipleAlignmentIO |  |
| ►Noverlaputils |  |
| CGeneSetIntersect |  |
| ►Nparser |  |
| CCommandLineParser |  |
| CStringParser |  |
| ►Nprimer3 |  |
| CComputeMIRScore |  |
| CComputeOriginalScore |  |
| CCrossJunctionPCRDesigner |  |
| CHairpinKmer |  |
| CisPCRLike |  |
| CPrimer3Exception |  |
| CPrimer3PickingSummary |  |
| CPrimer3PrimerPickingResult |  |
| CPrimerUtils |  |
| CRestrictionEnzyme |  |
| ►Nsequence |  |
| CAbstractFastaParser |  |
| CExtractor |  |
| CFastaHandler |  |
| CFastaParser |  |
| CFastaQualityBinaryIO |  |
| CFastaQualityHandler |  |
| CFastaQualityIndex |  |
| CFastaQualityParser |  |
| CFastaSequenceIO |  |
| CSequence |  |
| CSequenceRegion |  |
| CSequenceRegionWindow |  |
| CSequenceUtils |  |
| CTranscribedRegions |  |
| CTranscribedSequenceComposition |  |
| CWindowSlider |  |
| ►Nsiphy |  |
| ►Ntools |  |
| ►Nconservation |  |
| CAverageBranchLength |  |
| CBatchSubmitEstimateExon |  |
| CComputeKmersForRegion |  |
| CComputeMaxPercentileOfElements |  |
| CComputePercentageOfKmers |  |
| CEstimateOmegaPerExon |  |
| CSplitFile |  |
| CConservationUtils |  |
| CConservedPWMScanner |  |
| CEvolutionaryModel |  |
| CEvolutionaryModelParameters |  |
| CFit |  |
| CPhastconsReader |  |
| CPiHMM |  |
| CPositionWeightMatrixModel |  |
| CScaledWindowParserListener |  |
| CStationaryDistributionIO |  |
| CTreeScaler |  |
| CTreeScalerIO |  |
| CUnableToFitException |  |
| ►Nutil |  |
| CChromosomeStringComparator |  |
| CCLUtil |  |
| CCollapseByIntersection |  |
| CGMTParser |  |
| CParseGCTFile |  |
| ▼Npda |  |
| ►Nannotation |  |
| CBEDFileParser |  |
| CGTFFileParser |  |
| CLocus |  |
| ►Ncountreads |  |
| CAnnotationCounts |  |
| CFastqLibraryStats |  |
| ►Ndatastructures |  |
| CAlignments |  |
| CAlignmentsReader |  |
| CLocationAwareMatrix |  |
| ►NdifferentialExpression |  |
| CBatchNorm |  |
| CCisVsTrans |  |
| CCollapseProjections |  |
| CConvertToDiscreteMatrix |  |
| CDifferentialExpression |  |
| CDifferentialScoring |  |
| CFixedControlDifferentialExpression |  |
| CFoldSignificance |  |
| CFWERDistribution |  |
| CGrabProbes |  |
| CGSAProjection |  |
| CMakePositionalGeneSets |  |
| CMergeGSEAOutputIntoMatrix |  |
| CPermutations |  |
| CRunAllGSEABatch |  |
| CRunDifferentialExpression |  |
| CRunGSEA |  |
| CRunTimeSeries |  |
| CSplitByName |  |
| ►Nenrichment |  |
| CEnrichmentMetric |  |
| CEnrichmentUtils |  |
| ►Nfeature |  |
| ►Ngenome |  |
| CChromosome |  |
| CDirectoryInstalledGenomeAssembly |  |
| ►Ngene |  |
| CfunctionalAnnotation |  |
| CGeneTools |  |
| CGeneWithIsoforms |  |
| CNeighborAnalysis |  |
| CNeighborsNullModel |  |
| CtranscriptsNullModel |  |
| ►Ngeneexpression |  |
| ►Nagilent |  |
| ►Nanalysis |  |
| CProcessDataset |  |
| CAgilentArrayStats |  |
| CAgilentProbe |  |
| CAgilentToGCT |  |
| CAgilentUtils |  |
| CClassifyAgilentLincProbes |  |
| CCollapseProbesByMinPValue |  |
| CCorrelationAgilentNanostring |  |
| CCorrelationBetweenReplicates |  |
| CExtractSamplesAndLabel |  |
| CFilterMatrixByMinCutoff |  |
| CFindProbesForGeneSet |  |
| CMakeCLSFiles |  |
| CMakeCLSFromDescription |  |
| CQCAgilentResults |  |
| ►Nclustering |  |
| CAbsolutePearsonFunction |  |
| CCluster |  |
| CClusterDistanceFunction |  |
| CComputeCorrelationOfReplicates |  |
| CCorrelationMatrix |  |
| CEuclideanDistance |  |
| CHierarchicalClustering |  |
| CKmeans |  |
| CPearsonDistance |  |
| CRankOrder |  |
| CComputeHypergeomtricEnrichment |  |
| CCountNums |  |
| CExpressionExperimentInfo |  |
| CFilterGCT |  |
| CFilterGCTFile |  |
| CgeneExpUtils |  |
| CGlobalStats |  |
| CGrabSpecificColumns |  |
| CPaGEJava |  |
| CPreprocessDataSet |  |
| CScoreDiffGenes |  |
| ►Nrap |  |
| ►Nmisc |  |
| CFindEnrichedDepletedRegions |  |
| ►Nrnai |  |
| CExtractSequence |  |
| CRNAiGeneAnnotation |  |
| ►Nsamtools |  |
| CFilterFastqReads |  |
| CFilterPairedReadsByMAQ |  |
| CPairedEndReadIterator |  |
| CPairedEndTDF |  |
| CSAMFragmentWriter |  |
| CSAMPairedEndFileReader |  |
| CTrimFastqReads |  |
| CWrappedIterator |  |
| ►Nseq |  |
| ►Nalignment |  |
| CAlignmentUtils |  |
| CStoredAlignmentStats |  |
| ►Nchip |  |
| CComputeEnrichments |  |
| CFindDiffrentialEnrichedRegions |  |
| CPeakFilter |  |
| ►Nclip |  |
| CFastaExtendPeaks |  |
| CGenomeSpaceSampleData |  |
| CPileup |  |
| CSampleData |  |
| ►Nfastq |  |
| CFastqParser |  |
| CFastqSequence |  |
| CFastqSplitterByBarcode |  |
| CSplitAndTrimIndex |  |
| CSplitFastqByIndex |  |
| ►Ngraph |  |
| ►CChromosomeWithBubblesJGraphT |  |
| CEdgeSourceType |  |
| CPath |  |
| ►Nprotection |  |
| CBatchedMultiSampleScanPeakCaller |  |
| CGenomeSpaceSampleData |  |
| CMultiSampleScanPeakCaller |  |
| CReadSimulator |  |
| CReadSimulator2 |  |
| CSampleData |  |
| CTwoSampleScanSkellamPeakCaller |  |
| CWindowScores |  |
| ►Nrap |  |
| ►Nrna |  |
| CCollectRnaSeqMetrics |  |
| CCountReads |  |
| CTranscriptomeCommandLineProgram |  |
| CTranscriptomeScoringProgram |  |
| CBuildRatioNullDistribution |  |
| CCalculateCoverageStats |  |
| CCollectAnnotationEnrichments |  |
| CCountProbes |  |
| CCountReads |  |
| CDistanceToBED |  |
| CGCContent |  |
| CGenomeCommandLineProgram |  |
| CGenomeScoringProgram |  |
| CPlotAggregateRegions |  |
| CRAPPeakCaller |  |
| CRatioPermutationPeakCaller |  |
| CShuffleBAM |  |
| CShuffleBED |  |
| CSkellamKLPeakCaller |  |
| CSkellamScore |  |
| CSlideAndCalculate |  |
| CSlideAndCount |  |
| CUnmarkDuplicates |  |
| ►Nsegmentation |  |
| ►Nutils |  |
| CReconstructionComparer |  |
| CAlignmentDataModel |  |
| CAlignmentDataModelStats |  |
| CContinuousDataAlignmentModel |  |
| CDefineSourceAndSinkFromPairedEnds |  |
| CGeneCounts |  |
| CGenericAlignmentDataModel |  |
| CGeneScore |  |
| CGenomeWithGaps |  |
| CGenomeWithGaps2 |  |
| CGlobalSpliceFilter |  |
| CPrecomputeDataAlignmentStats |  |
| CReadCountNormalizer |  |
| CReadFilter< T > |  |
| CWrappedIGVIterator |  |
| ►Ntools |  |
| CPSLXToSAM |  |
| ▼Nprojection |  |
| ►Ngsa |  |
| CDefineGeneSignatures |  |
| CExtractGenes |  |
| CGeneSetEnrichment |  |
| CGSAScoring |  |
| CGsaUtils |  |
| ▼Njaligner |  |
| ▼Nformats |  |
| CCLUSTAL |  |
| CFASTA |  |
| CFormat |  |
| CFormatFactory |  |
| CPair |  |
| ▼Nmatrix |  |
| CMatricesCompartor |  |
| CMatrix |  |
| CMatrixGenerator |  |
| CMatrixLoaderException |  |
| ▼Nutil |  |
| CCommons |  |
| CSequenceParser |  |
| CSequenceParserException |  |
| CAlignment |  |
| CCell |  |
| CDirections |  |
| CMarkups |  |
| CNeedlemanWunsch |  |
| CNeedlemanWunschGotoh |  |
| CSequence |  |
| CSmithWatermanGotoh |  |
| CTracebackNode |  |
| ▼NJama |  |
| ▼Nutil |  |
| CMaths |  |
| CCholeskyDecomposition |  |
| CEigenvalueDecomposition |  |
| CLUDecomposition |  |
| CMatrix |  |
| CQRDecomposition |  |
| CSingularValueDecomposition |  |
| ▼Nnextgen |  |
| ▼Ncore |  |
| ►NreadFilters |  |
| CGenomicSpanFilter |  |
| CPairedAndProperFilter |  |
| CReadsToReconstructFilter |  |
| ▼Numms |  |
| ▼Ncore |  |
| ►Nalignment |  |
| CAbstractPairedEndAlignment |  |
| CAlignment |  |
| CAlignmentPair |  |
| CChromosomeInconsistencyException |  |
| ►CFeatureSequenceAlignment |  |
| CUnorderedGenePair |  |
| CFragmentAlignment |  |
| CPairedEndAlignmentFactory |  |
| CPairedReadAlignment |  |
| ►CSingleEndAlignment |  |
| CWrapSamRecord |  |
| CSmatchLike |  |
| CSmithWatermanAlignment |  |
| ►Nannotation |  |
| ►Nfilter |  |
| CFullyContainedFilter |  |
| COverlapFilter |  |
| CAbstractAnnotation |  |
| CAbstractAnnotationCollection< T extends Annotation > |  |
| ►CAnnotation |  |
| CStrand |  |
| CAnnotationCollection< T extends Annotation > |  |
| CAnnotationFileReader |  |
| CAnnotationList< T extends Annotation > |  |
| CBasicAnnotation |  |
| CCompoundInterval |  |
| CGene |  |
| CPSLRecord |  |
| CSingleInterval |  |
| ►Ncoordinatesystem |  |
| CAnnotationOutOfBoundsException |  |
| CCoordinateSpace |  |
| CGenomicSpace |  |
| CMaskedGenomicSpace |  |
| CPermutationNotFoundException |  |
| CShuffledIterator< T extends Annotation > |  |
| CTranscriptInGenomicSpace |  |
| ►CTranscriptomeSpace |  |
| CGeneTree |  |
| ►Nexception |  |
| CIncompleteMethodImplementationException |  |
| CRuntimeIOException |  |
| ►Nfeature |  |
| CGeneWindow |  |
| CGenomeWindow |  |
| CWindow |  |
| ►Ngeneral |  |
| CCloseableFilterIterator< T > |  |
| CPredicates |  |
| ►CTabbedReader |  |
| CFactory< T > |  |
| ►Nmodel |  |
| ►Nscore |  |
| CAbstractListScore |  |
| CBasicScanStatisticScore |  |
| CBinomialEnrichmentScore |  |
| CCountScore |  |
| CCoverageScore |  |
| CEndInWindowCountScore |  |
| CLengthScore |  |
| CMultiScore |  |
| CNewRatioScore |  |
| CPoissonEnrichmentScore |  |
| CRatioScore |  |
| CScanStatisticScore |  |
| CStartInWindowCountScore |  |
| CSumScore |  |
| CWindowAllScore |  |
| ►CWindowProcessor< S extends WindowScore > |  |
| CAbstractProcessor< S extends WindowScore > |  |
| ►CWindowScore |  |
| CAbstractWindowScore |  |
| CWindowScoreIterator< T extends WindowScore > |  |
| ►CAlignmentModel |  |
| CAlignmentCount |  |
| CFilteredIterator |  |
| CNodeIterator |  |
| CWrapAlignmentCountIterator |  |
| ►CJCSAlignmentModel |  |
| CAlignmentCount |  |
| CFilteredIterator |  |
| CNodeIterator |  |
| CWrapAlignmentCountIterator |  |
| ►Nreaders |  |
| CPairedEndReader |  |
| ►NreadFilters |  |
| CMappingQualityFilter |  |
| CPairedEndFilter |  |
| CPCRDuplicateFilter |  |
| CReadEndsInFilter |  |
| CReadStartsInFilter |  |
| CSameOrientationFilter |  |
| CSplicedReadFilter |  |
| CUniqueMappedReadsFilter |  |
| ►Nscripture |  |
| ►Nstatistics |  |
| CConnectDisconnectedTranscripts |  |
| CBuildScriptureCoordinateSpace |  |
| COrientedChromosomeTranscriptGraph |  |
| ►Nutils |  |
| CAnnotationUtils |  |
| ►Nwriters |  |
| CPairedEndWriter |  |
| ▼Nesat |  |
| CCollapsedGeneLengths |  |
| CESAT |  |
| CESATDistanceCalculator |  |


---

Generated on Fri Aug 15 2014 15:11:16 for ESAT by  

 1.8.7
